# A Cross-Entropy Approach to the Domination Problem and Its Variants

**DOI:** 10.3390/e26100844

**Published:** 2024-10-06

**Authors:** Ryan Burdett, Michael Haythorpe, Alex Newcombe

**Affiliations:** College of Science and Engineering, Flinders University, 1284 South Road, Tonsley Park, SA 5042, Australia; ryan.burdett@flinders.edu.au (R.B.); alex.newcombe@flinders.edu.au (A.N.)

**Keywords:** domination, total domination, 2-domination, secure domination, cross-entropy, variants, graphs

## Abstract

The domination problem and three of its variants (total domination, 2-domination, and secure domination) are considered. These problems have various real-world applications, including error correction codes, ad hoc routing for wireless networks, and social network analysis, among others. However, each of them is NP-hard to solve to provable optimality, making fast heuristics for these problems desirable. There are a wealth of highly developed heuristics and approximation algorithms for the domination problem; however, such heuristics are much less common for variants of the domination problem. We redress this gap in the literature by proposing a novel implementation of the cross-entropy method that can be applied to any sensible variant of domination. We present results from experiments that demonstrate that this approach can produce good results in an efficient manner even for larger graphs and that it works roughly as well for any of the domination variants considered.

## 1. Introduction

The graph domination problem has been studied for the best part of a century but gained additional interest in the 1970s when the closely related dominating set problem (for some given constant *K*, determining whether a dominating set with fewer than *K* vertices exists) was shown to be NP-complete [1]. Hence, the domination problem is NP-hard, and there is no known efficient algorithm which is guaranteed to be able to solve it. Despite this, applications in information networks and social networks and various graph-based operation research scenarios have necessitated the development of a number of algorithms for the domination problem [2,3,4]. The best exact algorithms have an exponential solving time, such as the $O(1.4969^{n})$ algorithm by van Rooij et al. [5], which determines not only the domination number but also the number of minimum dominating sets. The domination problem can also be formulated quite easily as a binary integer programming problem, which software such as CPLEX running on modern desktops is capable of solving, even for instances with hundreds of vertices. Beyond exact algorithms, there are several approximation algorithms [6,7] and fast heuristics [8,9,10]. A large portion of the experimental literature has focused on benchmarking algorithms that leverage the specific properties of domination.

In this paper, we add to this literature by adapting the cross-entropy (CE) method to the domination problem. CE was first proposed in 1997 by Rubinstein as a means of solving rare event probability estimation based on the method from [11]. It was subsequently expanded to solve combinatorial optimisation problems [12] and has been successfully applied to various NP-hard graph problems in particular, such as the traveling salesman problem [13] and the Hamiltonian cycle problem [14]. However, to the best of the authors’ knowledge, it has not previously been applied to the domination problem.

In addition to the domination problem, we will also show that the general approach we describe in this paper can be adapted to variants of the domination problem. There are many such variants of the domination problem, which typically arise because there is a real-world application for which the (standard) domination problem is not quite suitable. Unlike the domination problem, very few heuristics have been developed and investigated for these variants, and new variants (or combinations of existing variants) are regularly introduced in the literature. As such, it is desirable to have an approach which is adaptable to different variants without it needing to be redesigned or fine-tuned. To demonstrate the adaptability of our approach, we will apply it to three prominent variants of domination in particular.

The remainder of this paper is laid out as follows. In Section 2, we give some preliminary definitions and describe the variants of the domination problem that we will consider. In Section 3, we describe our implementation of the CE method. In Section 4, we provide experimental results showing the performance of our implementation. Finally, we make some concluding remarks in Section 5.

## 2. Preliminaries

We consider simple graphs *G* which contain a vertex set *V* and an edge set *E*, where n=|V| and m=|E|. If an edge uv∈E, we say that *u* and *v* are adjacent.

**Definition** **1** (Open neighbourhood and closed neighbourhood)**.** *Consider a graph G containing vertex set V and edge set E and a vertex v∈V. Then, N(v):={w|vw∈E} is called the open neighbourhood of v, and N[v]:=N(v)∪{v} is called the closed neighbourhood of v.*

Informally, we say that the open neighbourhood of *v* is the set of vertices which are adjacent to *v*. We will be primarily concerned with solving the domination problem, as defined in the following statements.

**Definition** **2** (Dominating set)**.** *A subset of the vertices S⊆V is a dominating set for G if every vertex in V∖S is adjacent to one or more vertices in S. That is, for every v∈V, we find N[v]∩S≠∅.*

**Definition** **3** (Domination problem and domination number)**.** *The domination problem for a graph G is determining the minimum cardinality among all dominating sets in G. The latter is denoted as γ(G) and is known as the domination number of G.*

### Variations of the Domination Method

We will now describe the three variants of dominating sets that will be considered in this paper.

Consider a graph *G* containing vertex set *V* and edge set *E* and a subset of the vertices S⊆V.

**Definition** **4** (Total dominating set)**.** *S is a total dominating set for G if every vertex in V is adjacent to one or more vertices in S.*

**Definition** **5** (2-dominating set)**.** *S is a 2-dominating set for G if every vertex in V∖S is adjacent to two or more vertices in S.*

**Definition** **6** (Secure dominating set)**.** *S is a secure dominating set for G if S is a dominating set, and for every vertex v∈V∖S, a vertex w∈S exists such that vw∈E, and (S∖{w})∪{v} is a dominating set.*

**Definition** **7** (Total (2-, secure) domination problem)**.** *The total (2-, secure) domination problem for a graph G is determining the minimum cardinality among all total (2-, secure) dominating sets in G. The latter is denoted as $\gamma_{t}$ ($\gamma_{2}$, $\gamma_{s}$) and is known as the total (2-, secure) domination number.*

As with the domination problem, some different algorithms have been developed for each of these variants, although the majority of these have been focused on solving particular kinds of graphs. For general graphs, there are few results. Burger et al. [15] showed that several variants of the domination problem (including total and secure domination) can be formulated as binary programming problems, which can then be solved to optimality using a solver such as CPLEX. It is easy to adapt such formulations to produce a similar binary programming formulation for 2-domination. Foerster [16] proposed an approximation algorithm for *k*-domination, while Chlebík and Chlebíková [17] showed that an approximation algorithm for minimum set cover can be adapted to provide an approximation algorithm for total domination. Unfortunately, the literature on variants of the domination problem is sprawling and often esoteric, typically combining several variants at once to produce results applicable to only very specific situations. This makes it challenging to identify existing algorithms for a desired specific variant. This, in large part, is the motivation for the present work; we seek to propose a framework that can be applied to essentially any variant of domination without requiring that variant to be analysed individually.

For more information on the three variants of domination considered in this paper, we refer interested readers to Henning and Yeo’s excellent book on total domination from 2013 [18], the 2012 survey on k-domination (and k-independence) by Chellali et al. [19], and the chapter detailing the state of the literature on secure domination (and eternal domination) by Klostermeyer and Mynhardt [20].

## 3. The Cross-Entropy Method

Cross-entropy is a function which compares the similarity of two probability distributions and, because of its comparative uses, has found diverse applications in statistics and machine learning. In information theory, cross-entropy serves as a measure for the cost (or efficiency) of using an incorrect model to make predictions compared to using the true model. Although cross-entropy has value in many applications, for our purposes, we are primarily interested in the so-called cross-entropy method, which is a Monte Carlo method that can be used to solve optimisation problems. It does this by repeating two phases; first, sample solutions are drawn from a given probability distribution, and the best solution found so far is recorded. Second, the probability distribution is updated by minimising the cross-entropy between the current probability distribution and some ideal target distribution. This process is continued until certain convergence parameters are met, at which time the best solution found is returned.

Although the cross-entropy method was first developed for rare event simulation, it can be applied to discrete optimisation naturally. We begin by setting constant values for some parameters N,M,ρ,α, and *r*, the meanings of which will become clear in the following explanation. Then, at iteration t≥0, a fast heuristic is used to generate *N* valid solutions according to a probability vector $P^{t}$, where we define $P^{0}$ to be a uniform probability vector. The solutions are then ranked by some appropriate scoring function, and we discard all but the best *M* solutions, which we call the elite set. A new probability vector $P^{*}$ corresponding to the elite set is computed; there are various ways to compute it, which we will discuss shortly, and the parameter ρ is used in this calculation to avoid numerical errors. Finally, we compute $P^{t+1}=\alpha P^{*}+\left(1-\alpha \right)P^{t}$ and proceed to the next iteration. Throughout this process, we keep track of the best solution found so far, and the algorithm terminates once *r* consecutive iterations have completed without any improvement in the best solution, returning this best solution as the final output. This approach is summarised in Algorithm 1.

There are various ways of using the elite set to compute $P^{*}$, and we will briefly summarise the simplest of these now. Suppose that we are using the cross-entropy method for a discrete optimisation problem that involves selecting a subset from some universe set satisfying the conditions of the underlying problem. For example, in the context of the domination problem, we need to select a subset of the vertices from the full vertex set so that the selected subset is dominating. Then, each entry in $P^{*}$ corresponds to an element from the universe set, and the purpose of $P^{*}$ is to give a higher probability to entry *i* if this entry appeared more often in the elite set. Hence, the simplest way of computing $P^{*}$ is to define $P_{i}^{*}$ as equal to the proportion of solutions from the elite set that contain entry *i*. Afterwards, we normalise $P^{*}$ to turn it into a probability vector.


**Algorithm 1** Cross-entropy method **Input**: Initial parameters *N*, *M*, ρ, α, and *r* **Output**: Minimum cardinality among the solutions found (best)  Set initial uniform probability vector $P^{0}$ best←∞ t←0 **while** best=∞, or best was updated within the past *r* iterations, **do**     Generate *N* solutions using $P^{t}$     Calculate the vector *L* of the scores for each solution     Sort the solutions and select the best *M* solutions as the elite
set     **if** min(L)<best, **then**         best←min(L)     **end if**     Calculate $P^{*}$ using the elite
set and ρ     $P^{t+1}\leftarrow \alpha P^{*}+\left(1-\alpha \right)P^{t}$     t←t+1 **end while**  **return**
 best


Of course, there are more sophisticated ways to compute $P^{*}$. For instance, rather than simply considering the proportion of solutions from the elite set containing an entry *i*, we can take into account the scores of those solutions, affording a stronger probability to the entries that appear in the higher-quality solutions. It is common to use inverse exponentials for this purpose, and this can sometimes result in some numerical issues on machines if high-precision numbers are not used. The parameter ρ is hence employed in the calculations to avoid the worst of these numerical issues. This is the approach that we will use, as will be explained in the following subsection.

### 3.1. Adapting the Cross-Entropy Method to the Domination Problem

For the domination problem, the universe set in question is the set of vertices in the graph. We can trivially assign a score to any dominating set by setting it as equal to the size of the set, with smaller scores being preferable. We will use the notation L(S)=|S| to denote the score of a dominating set *S*.

Recall that at each iteration, we will have generated an elite set of the *M* best solutions found, that is, the *M* (out of *N*) dominating sets of the lowest cardinality found. We denote this set by E. Furthermore, we will use the notation $E^{i}$ to refer to the subset of E that contains only those dominating sets that include the vertex *i*. Then, we will calculate $P^{*}$ using the following formula for each entry $P_{i}^{*}$, where $\delta :=\min_{S\in E}\frac{L(S)}{\log(\rho )}$.

$$P_{i}^{*}=\frac{\sum_{S\in E^{i}}e^{\frac{-L(S)}{\delta }}}{\sum_{S\in E}e^{\frac{-L(S)}{\delta }}}$$

Then, all that remains is to address how the dominating sets can be generated from a probability vector $P^{t}$. The process we advocate is as follows. We begin with a set *S*, which is initially empty, and make a copy of $P^{t}$, which we denote by $P^{†}$. Then, we select a vertex by making an observation of a discrete random variable with the probability mass function given by $P^{†}$ and add the selected vertex, say *v*, to *S*. Then, we check whether *S* is dominating. If not, we set $P_{v}^{†}=0$, normalise $P^{†}$ again, and repeat the process. It is clear that this process will eventually terminate; in the worst case, every single vertex will eventually be added to *S*, which will certainly be a dominating set.

Note that in the above paragraph, we described a process for generating a dominating set, but it is clear that an analogous process could be used for any desired variant of domination by simply checking the relevant domination criteria of that variant after each vertex is added. However, it is worth noting that for some variants of domination, the underlying graph may not contain any such sets. For instance, a graph with isolated vertices does not have any total dominating sets (in such a case, it is said that the total domination number is *∞*). For the variants that we consider in this paper, it is clear that every graph contains a dominating set, a 2-dominating set, and a secure dominating set. Hence, it is only when considering total dominating sets that we need to first consider the underlying graph.

Although the above approach will always generate a valid dominating set, there is nothing to ensure that the set generated this way is minimal. Since it is always beneficial to have minimal sets if possible, we augment the above approach with a second phase that iteratively considers each vertex *v* in *S* to see whether S∖{v} also satisfies the relevant domination criteria. If so, that vertex is removed from *S*, and the process continues until all vertices of *S* have been considered. It is clear that this process results in a minimal dominating set. The only question is in which order we should consider the vertices for removal; note that seeking to do so optimally is an NP-hard problem (to see this, note that *S* could contain every vertex at the conclusion of Phase 1, in which case solving the second phase optimally is equivalent to solving the domination problem). Instead, we propose the following fast heuristic. We again make a copy of $P^{t}$ and denote it as $P^{†}$. Then, we define $\bar{P}$ as the vector containing the following entries:

$${\bar{P}}_{i}:=\left\{\begin{array}{cc} 1-P_{i}^{†}, & \;\;\;\;\;\;\;\;\mathrm{if}\;i\in S,\\ 0 & \;\;\;\;\;\;\;\;\mathrm{otherwise}. \end{array}\right.$$

After normalising $\bar{P}$, we then use it in the same manner as we previously used $P^{†}$ to randomly generate a sequence of the vertices in *S*, and this is the order used to consider the vertices for removal. We summarise both phases in Algorithm 2, where the *rand* function corresponds to an observation of a discrete random variable with the probability mass function given by the associated probability vector.


**Algorithm 2** Algorithm for generating a minimal dominating set given $P^{t}$ **Input**: Graph *G*, probability vector $P^{t}$ **Output**: A minimal dominating set (*S*)  Phase 1: S←∅ $P^{†}\leftarrow P^{t}$ **while** *S* does not satisfy the relevant domination criteria for graph *G*, **do**     Normalise $P^{†}$.     $v\leftarrow rand(P^{†})$     S←S∪{v}     $P_{v}^{†}\leftarrow 0$ **end while**  Phase 2: $\bar{P}\leftarrow \left(1-P^{t}\right)$ **for** all vertices v∉S, **do**     ${\bar{P}}_{v}\leftarrow 0$ **end for** **while** $\bar{P}$ is not a zero vector, **do**     Normalise $\bar{P}$     $v\leftarrow rand(\bar{P})$     **if** S∖{v} satisfies the relevant domination criteria for graph *G*, **then**         S←S∖{v}     **end if**     ${\bar{P}}_{v}\leftarrow 0$ **end while**  **return** *S*


### 3.2. Checking the Relevant Domination Criteria

Note that for the cross-entropy implementation described in the previous subsections, we need to check the relevant domination criteria O(n) times for each of the *N* dominating sets generated per iteration. The standard method to check whether a set is dominating takes O(m) time, and so using this approach would require us to spend O(nmN) time each iteration to generate the dominating sets. This is the most computationally expensive component of the algorithm, and so we take steps to perform these checks more efficiently. Specifically, we utilise efficient updating procedures whenever we add or remove a vertex from *S* that allow us to track how close we are to meeting (or failing) the relevant domination criteria.

For (standard) domination, this is uncomplicated. For each vertex, we can keep track of how many vertices from its closed neighbourhood are in *S*. Whenever a vertex *v* is added to *S* (or removed from *S*), we need to update only those vertices adjacent to *v*. If we denote the degree of *v* as d(v), this updating procedure will occur in O(d(v)) time. Meanwhile, we can maintain a count of undominated vertices that is decreased whenever a vertex becomes dominated (or vice versa). Checking whether *S* is a dominating set is then as simple as checking whether this count is equal to zero. This approach requires us to spend O(mN) time per iteration, which represents an improvement by a considerable order of magnitude in the computation time. For 2-domination and total domination, checks analogous to the above are straightforward and provide similar improvements in the computation time.

Of the variants of domination considered in this paper, it is only secure domination that requires more thought. The naive method of checking whether *S* is secure dominating involves looking at each vertex *v* not in *S*, considering each of its neighbours *w* that are in *S*, and checking whether (S∪{u})∖{w} is dominating, which requires $O(m^{2})$ time. There are more sophisticated methods for checking secure domination. Burger et al. [21] give one such method; however, they do not indicate its computational complexity, other than to state that it is more complex than checking standard domination. Regardless, we again seek to improve on this by proposing an updating procedure. As before, for each vertex, we keep track of how many vertices in its closed neighbourhood are in *S*; for vertex *v*, we call this number domcount(v). Then, whenever a vertex *v* is added to or removed from *S*, we only need to update the vertices adjacent to *v*. Furthermore, we say that *w* is capable of defending *v* if w∈S∩N(v) and every neighbour of *w* is dominated in (S∪{v})∖{w}, and note that this criteria can be efficiently checked as follows. If there is any neighbour *u* of *w* such that domcount(u)=1 and u∉N[v], then *w* is not capable of defending *v*; otherwise, it is capable of defending *v*. Then, for each vertex, we keep track of how many vertices in its open neighbourhood are capable of defending it. Whenever a vertex *v* is added to *S* (or removed from *S*), we need to update only those vertices within a distance of 3 of *v*. Note that we can compute which vertices are within a distance of 3 of each vertex in advance (i.e., before the first iteration of cross-entropy begins), so this need not contribute to the runtime of the updating procedure. Finally, we can maintain a count of the vertices that are not in *S* and that have no vertices capable of defending them, and then *S* is a secure dominating set if and only if this count is equal to zero. For graph families where the diameter grows with *n*, this approach represents an improvement by some order of magnitude in the computation time over the course of generating the secure dominating set.

## 4. Experimental Results

### 4.1. Comparison Heuristics

Before beginning to describe the experimental setup and displaying the results of the cross-entropy approach, we will briefly pause to discuss how we will evaluate its performance.

The primary feature of the approach described in this manuscript is that it is very adaptable and can be applied to any variant of domination without requiring any significant redesign. Indeed, to adapt it to handle a new variant of domination, the only part of the algorithm that needs to be altered is the two lines in Algorithm 2 that check the relevant domination criteria. The latter can be modified entirely separately from the rest of the algorithm; indeed, one could imagine an implementation of this approach in which the core algorithm is fixed, and it is the user who provides their own function for checking the domination criteria relevant to them. This then allows any user who has interest in some new (or niche) variant of domination to access an effective heuristic for that variant without needing to invest time into designing one. If time is an obstacle (such as if the instances being considered are large or if it is inefficient to check the domination criteria), then the user can set the parameters accordingly to reduce the overall runtime and still be certain that they will be able to obtain a valid solution.

As such, although we are focusing on a few specific variants of domination in this manuscript, we do not find it meaningful to compare our results to the best-developed heuristics in the literature for these variants; naturally, one would expect that heuristics designed specifically for those variants to outperform a more general approach. However, since it is important to provide some point of comparison, we will compare the results to a greedy heuristic that is similarly flexible. Specifically, the greedy heuristic constructs a dominating set *S* of the desired variant by iteratively adding one vertex at a time, corresponding to the vertex whose closed neighbourhood contains the most vertices not yet in *S*. In the event that there are multiple candidates for *v*, we choose between them uniformly at random. In order to facilitate a fair comparison, we also equip this heuristic with an analogous version of Algorithm 2’s Phase 2, which ensures the provided dominating set is minimal. This greedy heuristic is summarised in Algorithm 3.


**Algorithm 3** Greedy heuristic for finding a minimal dominating set **Input**: Graph *G* **Output**: A minimal dominating set (*S*)  Phase 1: S←∅ **while** *S* does not satisfy the relevant domination criteria for graph *G*, **do**     v← the vertex such that |N[v]∖S| is largest (breaking ties uniformly at random)     S←S∪{v} **end while**  Phase 2: **for** each vertex v∈S (considered in a random order), **do**     **if** S∖{v} satisfies the relevant domination criteria for graph *G*, **then**         S←S∖{v}     **end if** **end for**  **return** *S*


### 4.2. Experimental Setup

The implementation of the cross-entropy method for domination described in Section 3 requires the user to input many parameters, and the overall performance (both in terms of solution quality and computation time) is impacted by these. In order to determine which combinations of parameters to use for the main experiments, initial experiments were conducted to identify good default values. The results of these experiments indicated that the following values provide a good balance between the solution quality and computation time for our implementation: N=100,M=10,α=0.2,ρ=0.01, and r=20. In particular, we found that increasing the amount of computation performed (i.e., by increasing the number of dominating sets generated per iteration or by increasing the number of iterations made without an improvement before stopping) from these default settings offered, at best, only marginal improvements in solution quality.

Using the above parameter settings, we now present the experimental results for our implementation of the cross-entropy method on several kinds of graphs. In particular, we consider grid graphs, flower snarks, unit disk graphs, Erdős–Rényi random graphs, and various graphs from the literature. The former two graph families have been chosen since the domination numbers (and some variants thereof) are known, while the remaining graphs have been chosen as they have previously been used to evaluate graph algorithms. Where possible, we obtain results for all four types of domination considered in this paper (domination, 2-domination, total domination, and secure domination). The only exception is for instances with isolated vertices, in which case we do not obtain results for total domination. We will now briefly discuss each type of graph we will consider.

Square grid graphs G(n,n) are the Cartesian products of two paths with the length *n*. They have been considered extensively in the context of domination, with the domination numbers now known for all cases [22]. Fascinatingly, the formula for the domination number in rectangular grid graphs G(m,n) contains 23 special cases before settling into a standard formula for m,n≥16. The values for the other variants of domination are also known in some cases [23,24,25].

Flower snarks J(k) are 3-regular graphs containing 4k vertices introduced by Isaacs [26]. In a recent paper [27], the domination, 2-domination, total domination, and secure domination numbers (among others) were determined.

Unit disk graphs [28] are generated in the following way. Given parameters c,r,m, and *n*, a set of *c* points are generated uniformly at random within an m×n rectangle. Then, a graph is produced where the vertices correspond to the points, and an edge exists between two vertices if the distance between their corresponding points is no more than 2r. Equivalently, one can draw circles of a radius *r* around each point, and then an edge exists between two vertices if their corresponding circles overlap. Of course, it is not guaranteed that such graphs will be connected; in Table 1, we label the graphs as “UDG_c-r-m-n_s”, where s corresponds to a random seed, and we only include graphs in our experiments that are connected.

Erdős–Rényi random graphs are generated in the following way. Given the parameters *N* and *p*, a graph with *N* vertices is generated in which each edge exists with probability *p*. In the interest of studying relatively sparse graphs, we have experimentally chosen values of *p* to ensure a low average degree for a given *N* and then generated many graphs with these parameter settings until a graph with an average degree very close to the desired value is obtained. As such, in Table 2, we label these graphs as “randomN_d” where d is the desired degree. Note that since these graphs have a low average degree, as *N* increases, it is almost certain that there will be some isolated vertices. As such, we do not consider total domination for these graphs.

Both unit disk graphs and Erdős–Rényi random graphs have been considered in many graph contexts, including as experimental instances for domination algorithms. For example, see [3,9], in which domination algorithms are presented. These papers also consider a number of other graphs from the literature, and so we have included many of them in our experiments as well, omitting only those which are too large to be computationally feasible. We have provided individual citations for the chosen instances in Table 3.

For each instance considered, we run the cross-entropy method ten times (with ten different random seeds) and then return the best solution produced. We compare this to the results obtained by the greedy heuristic given in Algorithm 3, which we also run ten times (with ten different random seeds) and return the best solution produced. Where possible, we will also compare the best solution produced by the cross-entropy method to the optimal solutions for those instances. For instances where the optimal solutions are not known from the literature, we use CPLEX to solve binary programming formulations of the relevant domination variant. In particular, we use the formulations for domination and total domination from [15] and the formulation for secure domination from [23]. We also use a formulation for 2-domination analogous to the formulation for domination in [15]. We set a time limit of 10,000 s for CPLEX to terminate, and if CPLEX is unable to produce an optimal solution by this time, we take the best solution (upper bound) produced up to this stage. The experiments were conducted on an Intel(R) Core(TM) i5-12500 CPU with a 6 core, a 3.00 GHz processor, and 16 GB of RAM, running Windows 10 Enterprise version 22H2 and using a C++ implementation of the cross-entropy algorithm. The solutions and upper bounds for the formulations were obtained on the same PC using CPLEX v22.1.0.

**Figure 1:**
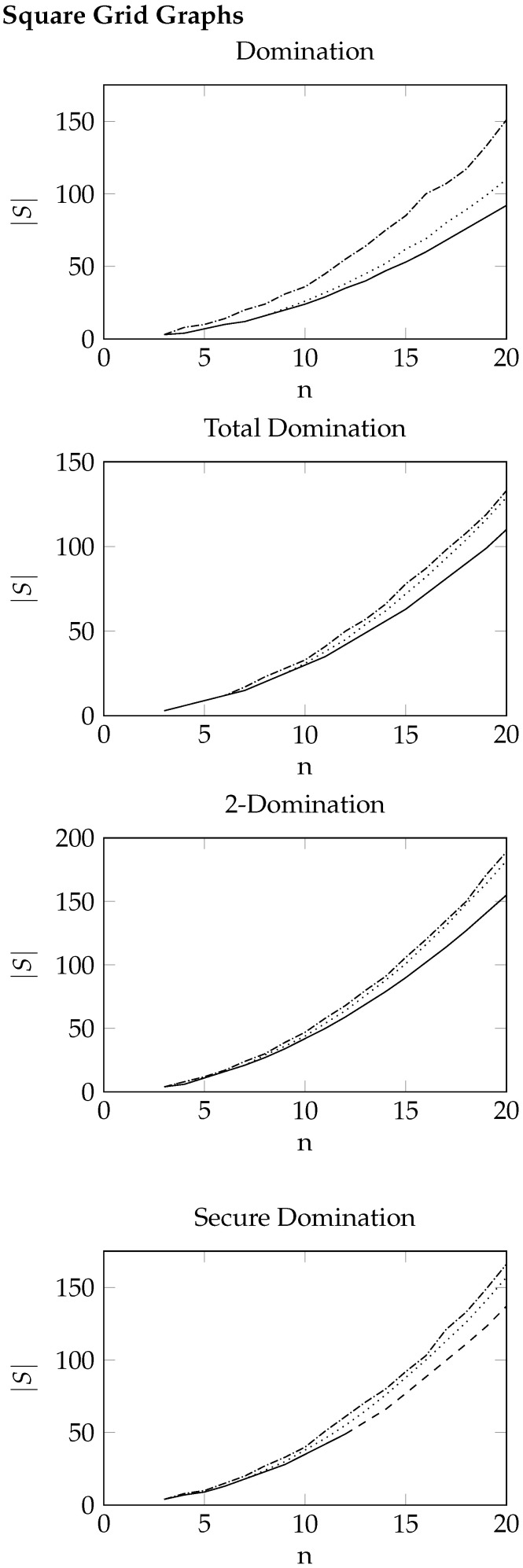
Results for square grid graphs G(n,n). The size of the best solutions returned by the cross-entropy method is displayed using a dotted line, while the size of the best solutions returned by the greedy heuristic is displayed using a dashdotted line. The known optimal values are displayed using a solid line. In the case of secure domination, a dashed line is used for the upper bounds obtained by CPLEX after 10,000 s.

**Figure 2:**
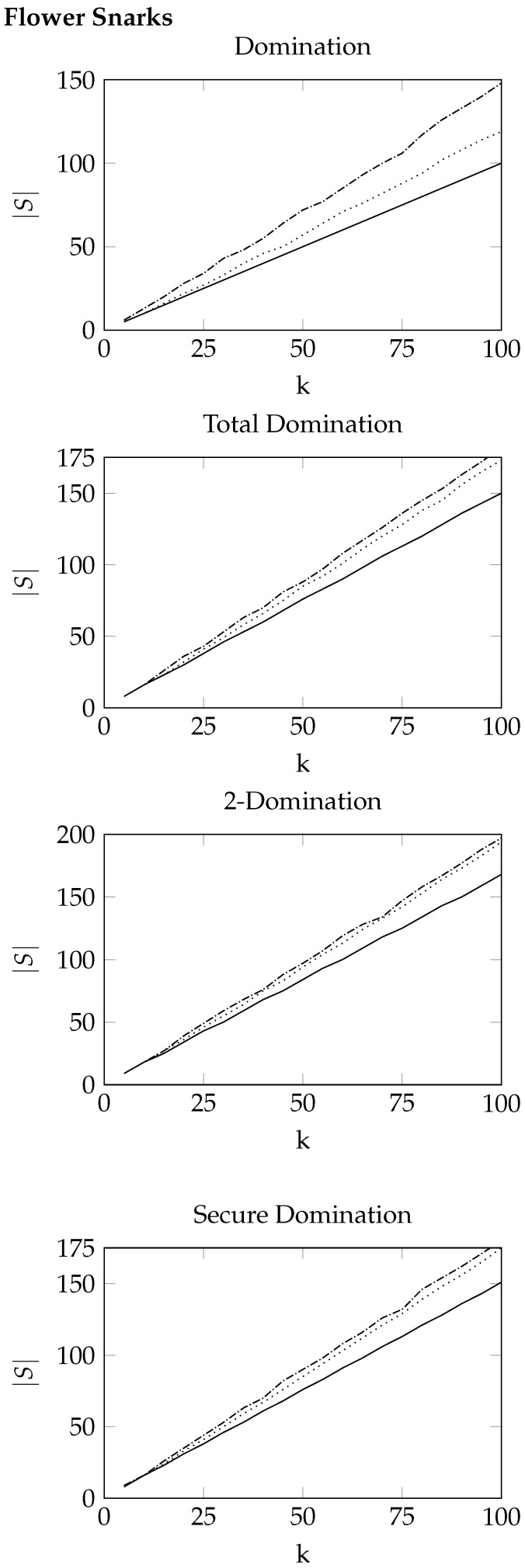
Results for flower snarks J(k). The size of the best solutions returned by the cross-entropy method is displayed using a dotted line, while the size of the best solutions returned by the greedy heuristic is displayed using a dashdotted line. The known optimal values are displayed using a solid line.

For square grid graphs and flower snarks, we present the results as plots since these graphs are not randomised and follow a set structure. In Figure 1 and Figure 2, the dotted lines represent the best values obtained from the cross-entropy method, the dashdotted lines represent the best values obtained from the greedy heuristic, and the solid lines represent the known optimal values. If the optimal values are not known, a dashed line is used to indicate the upper bound obtained by CPLEX after 10,000 s.

For the remaining graphs, the results are displayed in Table 1, Table 2 and Table 3. The “GH” column lists the best values obtained from the greedy heuristic, and the “CE” column lists the best values obtained from the cross-entropy method, while the “Sol” column lists either the optimal value (if it is known) or else the upper bound obtained by CPLEX after 10,000 s. In the latter case, the number is presented with an overline. Finally, the “Gap” column gives the difference between the values in the “CE” and “Sol” columns. If CPLEX was not even able to produce an upper bound within 10,000 s, we indicate this with a dash (-).

### 4.3. Results

We will first consider how the cross-entropy method compared to the greedy heuristic. In the vast majority of cases, the cross-entropy method produced better-quality solutions across all four variants, demonstrating its potency. However, for a small number of the largest graphs considered, the greedy heuristic did produce some superior solutions. This is most likely because the parameter settings we chose for the cross-entropy approach were fixed, regardless of the size of the instances.

Comparing the solutions found by the cross-entropy approach to the optimal solutions, it appears that this approach produces solutions of a roughly similar quality across all experiments; that is, for all kinds of graphs and variants of domination considered, it was generally able to find solutions within 10 to 25 percent of the known optimal solution or upper bound. However, certain variants of domination did occasionally perform slightly better for different graphs. For instance, secure domination performed relatively well for unit disk graphs, 2-domination performed relatively well for Erdős–Rényi random graphs, and total domination performed relatively well for the selected instances from the literature.

**Table 2:** Experimental results of the cross-entropy method for Erdős–Rényi random graphs for each domination variant except for total domination (since these graphs usually contain isolated vertices). The GH column shows the size of the best solutions returned by the greedy heuristic. The CE column shows the size of the best solutions returned by the cross-entropy method. The Sol column shows the cardinality of an optimal set for the given instance or the upper bound if the value is overlined. The Gap column shows the difference between the CE and Sol values. A dash is used when CPLEX was unable to even obtain an upper bound within 10,000 s.

Instance	Domination				2-Domination				Secure Domination			
	**GH**	**CE**	**Sol**	**Gap**	**GH**	**CE**	**Sol**	**Gap**	**GH**	**CE**	**Sol**	**Gap**
random100_3	40	35	35	0	60	58	58	0	51	48	47	1
random100_4	32	29	28	1	51	48	48	0	42	41	39	2
random100_5	27	24	23	1	44	41	40	1	36	36	33	3
random100_6	23	21	19	2	39	35	33	2	32	31	28	3
random250_3	91	87	81	6	140	138	131	7	119	115	105	10
random250_4	79	73	65	8	123	117	108	9	106	102	90	12
random250_5	67	62	53	9	110	103	92	11	96	91	-	-
random250_6	61	56	46	10	97	92	81	11	82	82	$\bar{71}$	11
random500_3	177	168	148	20	283	274	255	19	236	234	209	25
random500_4	149	141	117	24	241	228	203	25	209	202	$\bar{170}$	32
random500_5	122	121	98	23	211	200	$\bar{171}$	29	180	179	$\bar{147}$	32
random500_6	111	108	84	24	190	180	$\bar{151}$	29	168	162	$\bar{134}$	28
random800_3	295	280	250	30	465	450	419	31	398	387	344	43
random800_4	250	234	195	39	395	383	339	44	345	336	$\bar{282}$	54
random800_5	214	207	163	44	355	339	$\bar{290}$	49	309	299	$\bar{249}$	50
random800_6	190	184	$\bar{141}$	43	314	301	$\bar{250}$	51	266	272	$\bar{222}$	50
random1000_3	390	374	329	45	595	578	536	42	512	504	$\bar{449}$	55
random1000_4	320	310	256	54	511	495	438	57	437	434	$\bar{367}$	67
random1000_5	370	269	212	57	448	430	$\bar{366}$	64	391	382	$\bar{321}$	61
random1000_6	242	242	$\bar{183}$	59	403	391	$\bar{323}$	68	344	351	$\bar{285}$	66

**Table 3:** Experimental results of the cross-entropy method for the selected literature instances for each domination variant. The GH column shows the size of the best solutions returned by the greedy heuristic. The CE column shows the size of the best solutions returned by the cross-entropy method. The Sol column shows the cardinality of an optimal set for the given instance or the upper bound if the value is overlined. The Gap column shows the difference between the CE and Sol values. A dash is used when CPLEX was unable to even obtain an upper bound within 10,000 s. An *∞* symbol is used whenever the graph contains no total dominating set.

Instance	Domination				Total Domination				2-Domination				Secure Domination			
	**GH**	**CE**	**Sol**	**Gap**	**GH**	**CE**	**Sol**	**Gap**	**GH**	**CE**	**Sol**	**Gap**	**GH**	**CE**	**Sol**	**Gap**
adjnoun [29]	18	18	18	0	20	19	19	0	42	39	38	1	35	32	31	1
anna [30]	12	12	12	0	12	12	12	0	50	47	47	0	47	42	42	0
david [30]	2	2	2	0	2	2	2	0	27	26	26	0	24	24	24	0
dolphins [31]	14	14	14	0	17	17	17	0	29	27	27	0	22	22	22	0
football [32]	15	13	12	1	18	15	13	2	26	24	21	3	19	18	17	1
gplus_2000 [3]	174	236	170	66	191	188	181	7	1062	1036	965	71	955	949	-	-
gplus_500 [3]	42	42	42	0	48	45	45	0	315	303	297	6	284	274	267	7
homer [30]	97	97	96	1	*∞*	*∞*	*∞*		329	323	317	6	303	294	282	12
huck [30]	9	9	9	0	11	11	11	0	21	21	21	0	15	15	15	0
lesmis [33]	10	10	10	0	10	10	10	0	33	33	33	0	31	28	28	0
netscience [29]	535	509	477	32	∞	∞	∞		954	933	915	18	657	643	623	20
pokec_2000 [3]	75	78	75	3	76	75	75	0	921	879	816	63	871	853	-	-
pokec_500 [3]	16	16	16	0	16	16	16	0	280	266	264	2	270	257	251	6
polbooks [3]	15	14	13	1	16	15	15	0	27	24	22	2	21	21	19	2
power [34]	1584	1747	1481	266	1947	1932	1801	131	3047	3002	2795	207	2593	2575	-	-
zachary [35]	4	4	4	0	4	4	4	0	12	12	12	0	9	9	9	0

We highlight a few instances in particular. From Table 3, the gplus_2000 instance provided fascinating results. For (standard) domination, the best solution found for the cross-entropy approach had a gap of 66 from the optimal solution, and yet for total domination, the gap was only 7. Even for 2-domination, the gap is 71, but the size of the optimal 2-dominating set is more than five times as large as that of the optimal dominating set. Similarly, for pokec_2000, for (standard) domination, the best solution found had a gap of 3, but for total domination, an optimal solution was found which itself was also an optimal dominating set. It seems that in these more difficult instances, having a variant of domination can actually be beneficial; one possible explanation is that the more restrictive constraints of these variants force the cross-entropy algorithm to consider only certain types of dominating sets that are perhaps closer to the optimal solution on average. Indeed, comparing these results to the greedy heuristic, we see that the greedy heuristic was much more successful for (standard) domination than the cross-entropy method but was less successful for each of the variants.

**Figure 3:**
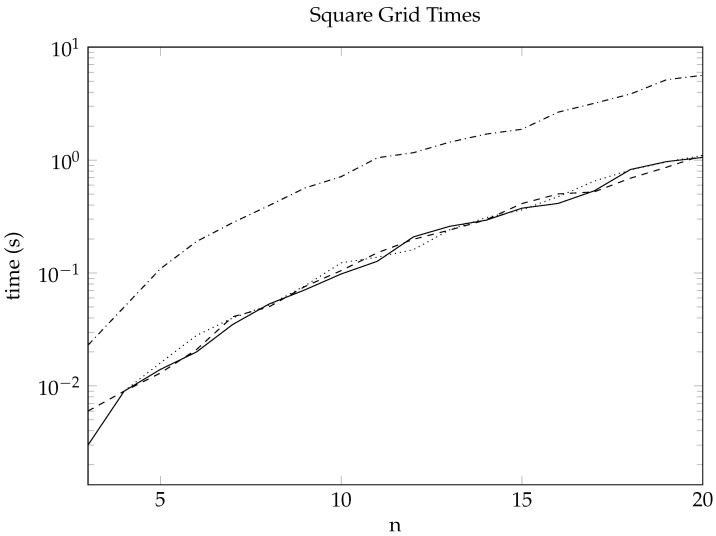
Average runtime in seconds for a single run of the cross-entropy algorithm on square grid graphs G(n,n) for the different variants of domination. The solid line corresponds to the domination problem; the dashed line to total domination; the dotted line to 2-domination; and the dashdotted line to secure domination.

In both the gplus_2000 and pokec_2000 instances, CPLEX was unable to produce even an upper bound for secure domination within 10,000 s. This highlights the value of having a heuristic at hand that can be implemented easily for any sensible variant of domination and can generate solutions of reasonable quality quickly. Across our experiments, we found that the order of the graph was the main factor affecting how long the cross-entropy algorithm took to run, with the edge structure of the graph being relatively unimportant. As such, in Figure 3, we provide the average runtimes just for the square grid graphs, with a very similar performance being exhibited for all other graphs.

As can be seen, the time taken for the cross-entropy algorithm to run is fairly consistent between domination, 2-domination, and total domination, while it is roughly an order of magnitude slower for secure domination. This demonstrates that the main factor in the overall computation time for our implementation is just the time taken to actually generate the sample solutions each iteration, with the remainder of the algorithm adding very little overhead per iteration. As such, it is imperative that the generation algorithms are optimised to ensure the overall algorithm can be run efficiently. We note here that even for graphs with 400 vertices, the cross-entropy algorithm was able to produce dominating sets, 2-dominating sets, and total dominating sets of reasonable quality in less than a second and secure dominating sets in less than ten seconds, demonstrating the efficiency of this algorithm. Indeed, by looking at Figure 1, we see that CPLEX was unable to find an optimal secure dominating set for the square grid graph G(13,13), and after 10,000 s of computation, it was only able to find an upper bound marginally better than that produced by the cross-entropy implementation in less than 2 s.

## 5. Conclusions and Future Work

Although there are a wealth of fast heuristics for the (standard) domination problem, its variants often have very few such heuristics available, and developing such heuristics typically requires one to first analyse the specific variant being considered. In contrast, the implementation of the cross-entropy method described in this paper can be applied easily to most variants of domination without requiring any modification, and the experiments in Section 4 indicate that the solution quality remains consistent for different variants, with it typically outperforming the greedy heuristic. In particular, our method is suitable for any variant of domination in which simply adding vertices randomly is guaranteed to result in a set meeting the criteria of the variant; or, if there are no such sets in a given graph, we should be able efficiently identify this in advance. Then, all that is required to use our method is to provide a checking algorithm that determines whether any given set meets the criteria of the desired variant.

The implementation itself is very lightweight outside of the checking algorithms, making it particularly suitable for large instances. Although it does not typically return an optimal solution for large instances, it is able to find good solutions very efficiently without the associated memory and performance issues that come with formulating the problem as a mixed-integer linear program or requiring any other analysis of the underlying problem. The checking algorithm itself may be sped up in a number of ways; for example, the domination criteria do not need to be checked after each vertex is added and can instead be checked at regular intervals, particularly given that Phase 2 of Algorithm 2 will trim unnecessary vertices out of the set. The checking algorithm may also be sped up using specialised hardware such as a GPU to distribute the workload in parallel. Since the checking algorithm is, by far, the slowest component of the algorithm, any improvements to it will correspond to a direct improvement in the overall computation time.

We can finish by noting that there are some variants of domination for which it is not the case that adding vertices randomly is guaranteed to result in a valid solution. For example, an independent dominating set is a dominating set that is also an independent set in the underlying graph, and adding vertices randomly could potentially violate the latter criterion. However, even in such cases, it may be possible to modify the cross-entropy implementation accordingly. For example, in the case of independent domination, upon adding a vertex to *S*, in addition to setting the probability of that vertex to 0, one can also set the probability of its neighbours to 0, and this will ensure that an independent dominating set is generated. 

## Figures and Tables

**Table 1:** Experimental results of the cross-entropy method for unit disk graphs for each domination variant. The GH column shows the size of the best solutions returned by the greedy heuristic. The CE column shows the size of the best solutions returned by the cross-entropy method. The Sol column shows the cardinality of an optimal set for the given instance or an upper bound if the value is overlined. The Gap column shows the difference between the CE and Sol values.

Instance	Domination				Total Domination				2-Domination				Secure Domination			
	**GH**	**CE**	**Sol**	**Gap**	**GH**	**CE**	**Sol**	**Gap**	**GH**	**CE**	**Sol**	**Gap**	**GH**	**CE**	**Sol**	**Gap**
UDG_100-0.7-10-10_17	21	19	19	0	25	24	24	0	36	35	34	1	28	27	26	1
UDG_100-0.7-10-10_32	19	18	18	0	26	25	25	0	37	35	34	1	28	27	27	0
UDG_100-0.7-10-10_43	21	20	20	0	31	28	27	1	40	38	36	2	27	28	27	1
UDG_100-0.7-10-10_55	21	20	20	0	26	24	24	0	39	36	36	0	27	27	27	0
UDG_100-0.7-10-10_73	22	20	20	0	30	27	26	1	39	38	36	2	29	28	27	1
UDG_500-0.4-10-10_0	69	65	55	10	86	85	71	14	129	125	106	19	97	94	81	13
UDG_500-0.4-10-10_1	68	66	55	11	86	85	72	13	127	124	104	20	97	96	82	14
UDG_500-0.5-10-10_0	47	44	37	7	60	58	46	12	88	85	71	14	68	67	$\bar{58}$	9
UDG_500-0.5-10-10_1	44	46	37	9	61	60	47	13	88	87	73	14	68	68	$\bar{58}$	10
UDG_800-0.3-10-10_0	119	118	99	19	159	154	124	30	222	217	183	34	165	166	$\bar{141}$	25
UDG_800-0.3-10-10_1	119	118	99	19	154	150	122	28	226	217	184	33	170	169	$\bar{144}$	25
UDG_800-0.5-10-10_0	51	48	39	9	62	63	$\bar{47}$	16	97	94	$\bar{74}$	20	75	74	$\bar{69}$	5
UDG_800-0.5-10-10_1	51	49	39	10	66	63	$\bar{48}$	15	97	95	$\bar{76}$	19	76	75	$\bar{69}$	6
UDG_1000-0.3-10-10_0	125	124	99	25	166	159	$\bar{123}$	36	227	227	$\bar{191}$	36	177	176	$\bar{149}$	27
UDG_1000-0.3-10-10_1	126	121	99	22	165	155	$\bar{121}$	34	234	229	$\bar{200}$	29	180	177	$\bar{152}$	25
UDG_1000-0.5-10-10_0	54	50	39	11	65	64	$\bar{51}$	13	99	97	$\bar{77}$	20	77	76	$\bar{75}$	1
UDG_1000-0.5-10-10_1	54	52	39	13	69	64	$\bar{50}$	14	96	98	$\bar{76}$	22	81	77	$\bar{71}$	6

## Data Availability

The set of instances analysed in this study and the results of that analysis are available in the GitHub repository https://github.com/flinders-maths/test_instances (accessed on 30 September 2024).

## References

[1] Garey M.R., Johnson D.S. (1979). Computers and Intractability.

[2] Dai F., Wu J. (2004). An extended localized algorithm for connected dominating set formation in ad hoc wireless networks. IEEE Trans. Parallel Distrib. Syst..

[3] Chalupa D. (2018). An order-based algorithm for minimum dominating set with application in graph mining. Inf. Sci..

[4] Corcoran P., Gagarin A. (2021). Heuristics for k-domination models of facility location problems in street networks. Comput. Oper. Res..

[5] Van Rooij J.M., Bodlaender H.L. (2011). Exact algorithms for dominating set. Discret. Appl. Math..

[6] Mira F.A.H., Inza E.P., Almira J.M.S., Vakhania N. (2022). A polynomial-time approximation to a minimum dominating set in a graph. Theor. Comput. Sci..

[7] Parekh A.K. (1991). Analysis of a greedy heuristic for finding small dominating sets in graphs. Inf. Process. Lett..

[8] Campan A., Truta T.M., Beckerich M. Fast Dominating Set Algorithms for Social Networks. Proceedings of the 26th Modern Artificial Intelligence and Cognitive Sciences Conference.

[9] Casado A., Bermudo S., López-Sánchez A., Sánchez-Oro J. (2023). An iterated greedy algorithm for finding the minimum dominating set in graphs. Math. Comput. Simul..

[10] Eubank S., Kumar V.A., Marathe M.V., Srinivasan A., Wang N. Structural and algorithmic aspects of massive social networks. Proceedings of the Fifteenth Annual ACM-SIAM Symposium on Discrete Algorithms.

[11] Rubinstein R.Y. (1997). Optimization of computer simulation models with rare events. Eur. J. Oper. Res..

[12] Rubinstein R. (1999). The cross-entropy method for combinatorial and continuous optimization. Methodol. Comput. Appl. Probab..

[13] De Boer P.T., Kroese D.P., Mannor S., Rubinstein R.Y. (2005). A tutorial on the cross-entropy method. Ann. Oper. Res..

[14] Eshragh A., Filar J.A., Haythorpe M. (2011). A hybrid simulation-optimization algorithm for the Hamiltonian cycle problem. Ann. Oper. Res..

[15] Burger A., De Villiers A., Van Vuuren J. A binary programming approach towards achieving effective graph protection. Proceedings of the 2013 ORSSA Annual Conference.

[16] Foerster K.T. Approximating fault-tolerant domination in general graphs. Proceedings of the 2013 Tenth Workshop on Analytic Algorithmics and Combinatorics (ANALCO).

[17] Chlebík M., Chlebíková J. (2008). Approximation hardness of dominating set problems in bounded degree graphs. Inf. Comput..

[18] Henning M.A., Yeo A. (2013). Total Domination in Graphs.

[19] Chellali M., Favaron O., Hansberg A., Volkmann L. (2012). k-domination and k-independence in graphs: A survey. Graphs Comb..

[20] Klostermeyer W.F., Mynhardt C.M. (2020). Eternal and Secure Domination in Graphs. Topics in Domination in Graphs.

[21] Burger A., De Villiers A., Van Vuuren J. (2013). Two algorithms for secure graph domination. J. Comb. Math. Comb. Comput..

[22] Gonçalves D., Pinlou A., Rao M., Thomassé S. (2011). The domination number of grids. SIAM J. Discret. Math..

[23] Burdett R., Haythorpe M. (2020). An improved binary programming formulation for the secure domination problem. Ann. Oper. Res..

[24] Rao M., Talon A. (2019). The 2-domination and Roman domination numbers of grid graphs. Discret. Math. Theor. Comput. Sci..

[25] Soltankhah N. (2010). Results on total domination and total restrained domination in grid graphs. Int. Math. Forum.

[26] Isaacs R. (1975). Infinite families of nontrivial trivalent graphs which are not Tait colorable. Am. Math. Mon..

[27] Burdett R., Haythorpe M., Newcombe A. (2023). Variants of the domination number for flower snarks. Ars Math. Contemp..

[28] Clark B.N., Colbourn C.J., Johnson D.S. (1990). Unit disk graphs. Discret. Math..

[29] Newman M.E. (2006). Finding community structure in networks using the eigenvectors of matrices. Phys. Rev. E.

[30] Johnson D.S., Trick M.A. (1996). Cliques, Coloring, and Satisfiability: Second DIMACS Implementation Challenge, October 11–13, 1993.

[31] Lusseau D., Schneider K., Boisseau O.J., Haase P., Slooten E., Dawson S.M. (2003). The bottlenose dolphin community of doubtful sound features a large proportion of long-lasting associations: Can geographic isolation explain this unique trait?. Behav. Ecol. Sociobiol..

[32] Girvan M., Newman M.E. (2002). Community structure in social and biological networks. Proc. Natl. Acad. Sci. USA.

[33] Knuth D.E. (1993). The Stanford GraphBase: A Platform for Combinatorial Computing.

[34] Watts D.J., Strogatz S.H. (1998). Collective dynamics of ‘small-world’networks. Nature.

[35] Zachary W.W. (1977). An information flow model for conflict and fission in small groups. J. Anthropol. Res..

